# The effect of contextual cues on the encoding of motor memories

**DOI:** 10.1152/jn.00773.2012

**Published:** 2013-02-27

**Authors:** Ian S. Howard, Daniel M. Wolpert, David W. Franklin

**Affiliations:** Computational and Biological Learning Lab, Department of Engineering, University of Cambridge, Cambridge, United Kingdom

**Keywords:** motor learning, visual cues, interference, dynamic adaptation, state-dependent learning

## Abstract

Several studies have shown that sensory contextual cues can reduce the interference observed during learning of opposing force fields. However, because each study examined a small set of cues, often in a unique paradigm, the relative efficacy of different sensory contextual cues is unclear. In the present study we quantify how seven contextual cues, some investigated previously and some novel, affect the formation and recall of motor memories. Subjects made movements in a velocity-dependent curl field, with direction varying randomly from trial to trial but always associated with a unique contextual cue. Linking field direction to the cursor or background color, or to peripheral visual motion cues, did not reduce interference. In contrast, the orientation of a visual object attached to the hand cursor significantly reduced interference, albeit by a small amount. When the fields were associated with movement in different locations in the workspace, a substantial reduction in interference was observed. We tested whether this reduction in interference was due to the different locations of the visual feedback (targets and cursor) or the movements (proprioceptive). When the fields were associated only with changes in visual display location (movements always made centrally) or only with changes in the movement location (visual feedback always displayed centrally), a substantial reduction in interference was observed. These results show that although some visual cues can lead to the formation and recall of distinct representations in motor memory, changes in spatial visual and proprioceptive states of the movement are far more effective than changes in simple visual contextual cues.

during interactions with objects in the environment, our sensorimotor control system uses sensory information to adapt to novel dynamics (Lackner and Dizio 1994; Shadmehr and Mussa-Ivaldi 1994). Two conceptually different forms of sensory information can be used. First, error-related sensory information about the dynamics, such as proprioceptive and visual errors, can be used to update the controller (Franklin et al. 2008; Kawato et al. 1987; Shadmehr et al. 2010; Thoroughman and Shadmehr 2000; van Beers 2009). Second, there are contextual sensory cues, which are not directly related to errors but can be informative about the dynamics. For example, the visual appearance of an object, such as whether it looks like it is made of metal or plastic, or the geometrical structure of an object can be used to adjust control in an anticipatory manner (Flanagan et al. 2008; Gordon et al. 1993; Ingram et al. 2010).

It is well known that in the absence of contextual information, two opposing dynamical force fields presented sequentially produce substantial interference during learning (Brashers-Krug et al. 1996; Caithness et al. 2004; Gandolfo et al. 1996; Karniel and Mussa-Ivaldi 2002; Krakauer et al. 1999). However, several studies have shown that when different sensory contextual cues are each associated with one of the fields, interference can be reduced (Addou et al. 2011; Cothros et al. 2009; Hirashima and Nozaki 2012; Howard et al. 2012; Hwang and Shadmehr 2005; Hwang et al. 2006; Krouchev and Kalaska 2003; Osu et al. 2004; Richter et al. 2003; Wada et al. 2003). This shows that contextual information can allow the formation and recall of separate motor memories for the opposing dynamics.

Although a range of contextual cues has been examined, because prior studies only used a small set of cues, often in a unique paradigm, it is hard to assess the conflicting results or determine the relative efficacy of different sensory contextual cues. Such differences in experimental design include the number of targets, the form and strength of the dynamic force fields, the ordering of trials, the use of arm movements vs. elbow movements, and the length of the movements. In particular, some studies have examined only a single movement direction (e.g., Hirashima and Nozaki 2012; Hwang et al. 2006), which raises a possible problem, since cognitive strategies may be easier to use for a single movement direction. There are also conflicting reports of the behavior of some cues in the literature. In particular, the effectiveness of color as a contextual cue for learning opposing dynamics is controversial. One of the first studies showed no reduction in interference when the room color lighting was linked to each field (Gandolfo et al. 1996). However, more recent studies have demonstrated a reduction in interference with extensive training both in monkeys (Krouchev and Kalaska 2003) and in humans (Addou et al. 2011) or where there is random and frequent switching between the two fields (Osu et al. 2004; Wada et al. 2003).

There is extensive evidence suggesting that motor memories can be learned as a function of limb state. For example, two opposing force fields can be learned if each is linked with a different grasp of the robot handle (Gandolfo et al. 1996) or with different locations in the workspace (Hwang et al. 2006). A strong effect of the location of visual feedback of the hand and targets also has been recently demonstrated (Hirashima and Nozaki 2012). In the study, participants made movements to two possible targets, one to the left and the other to the right of straight ahead. However, by associating a counterclockwise and a clockwise visuomotor rotation of the hand cursor with trials to the left and right targets, it was possible to get the subjects to make the same physical movement (i.e., straight ahead) to achieve different visual movements to the two targets. When each target was associated with a different field, participants could learn the opposing dynamics on the basis of the differences in visual feedback despite the movements being in the same physical space.

In the present study we use an interference paradigm to investigate the relative effectiveness of different contextual cues and changes in state, some of which have been investigated previously and some of which are novel. We distinguish state changes from contextual cues, with the latter referring to sensory information unrelated to the state of the arm. Therefore, cues can include features such as the background color or visual orientation of a handheld object. In contrast, state changes can include different configurations of the arm, whether real or perceived. However, it is possible for state changes to be investigated within the same framework as that used for sensory contextual cue, thereby unifying these conditions within the same experimental paradigm. Therefore, for convenience, we refer to the different experimental conditions, be they sensory cues or states, as contextual cues. Our unified interference paradigm ensures that each interference experiment differs only in terms of the contextual cue, with all other experimental features remaining consistent across experiments. This allows us to assess and rank their ability in the formation and recall of distinct motor memories. Subjects performed center-out reaching movements to eight targets in which one of two opposing curl force fields was applied. The direction of the curl field was consistent with a contextual cue, which was presented both before and during the movement. This paradigm was used to contrast the effectiveness of seven contextual cues: cursor color, background color, peripheral visual motion, object orientation, pure visual offset, pure movement offset, and workspace offset. Whereas some of these conditions have been studied previously, albeit many using only a single direction of movement, the peripheral visual motion and object orientation are novel conditions.

## MATERIALS AND METHODS

A total of 48 (22 male, 26 female) right-handed subjects, mean age 22.7 (SD 5.2) yr old, took part in 8 experiments (6 subjects in each experiment). Subjects provided written informed consent and were naive to the aims of the experiments. A local ethics committee approved the protocol, and all subjects were right-handed as based on the Edinburgh handedness questionnaire (Oldfield 1971).

### 

#### Apparatus.

**Fig. 1.**
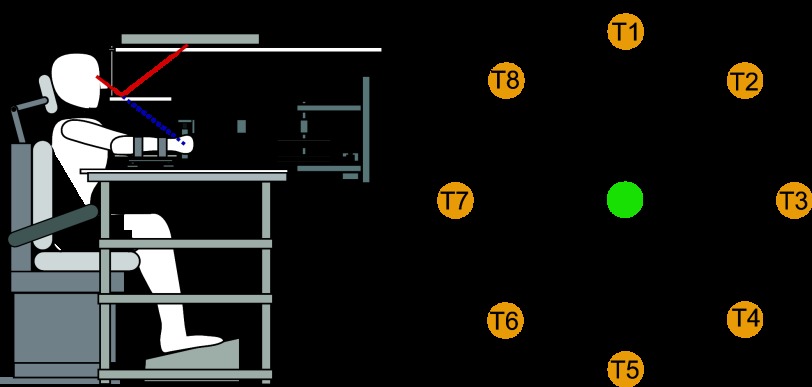
Experimental paradigm. *A*: the subject grasps the handle of the robotic manipulandum (vBOT) while seated. Visual feedback of movements is presented veridically using a horizontally mounted monitor viewed through a mirror. The subject's forearm is fixed to the handle and supported by an air sled. *B*: workspace layout of the experiment. There was a single starting location (green circle; note that in the experiment this was displayed as gray) and 8 targets (yellow circles: T1–T8).

All experiments were performed with the use of a vBOT planar robotic manipulandum, with associated virtual reality system and air table (Howard et al. 2009). The vBOT is a custom-built back-drivable planar robotic manipulandum, which exhibits low mass at its handle. Position is measured with optical encoders sampled at 1,000 Hz, and torque motors allow endpoint forces to be specified. The position signal was used unfiltered, whereas velocity was computed by fitting a quadratic motion equation, assuming constant acceleration, over a window that consisted of the 30 most recent position samples and associated time stamps. The vBOT was fitted with a force transducer (Nano 25; ATI) mounted at the handle to measure the applied forces. Before digitization, the output channels of the force transducers were low-pass filtered at 500 Hz using analog 4th-pole Bessel filters. Subjects were seated in a sturdy chair in front of the apparatus and firmly strapped against the backrest with a four-point seatbelt to reduce body movement (Fig. 1*A*). Subjects grasped the robot handle in their right hand while an air sled (constraining movement to the horizontal plane) supported their right forearm. Visual feedback was provided using a computer monitor mounted above the vBOT and was projected veridically to the subject via a mirror. This mirror prevented subjects from viewing their hand directly and allowed the visual feedback of the target (1.25-cm-radius disk), the starting location (1.0-cm-radius disk), and hand cursor (0.5-cm-radius red disk) to be presented in the plane of movement. In all experiments, except for the background color cue experiment, a black background was employed.

#### Paradigm overview.

In all the experiments, a trial consisted of a contextual cue associated with an adaptation movement. The adaptation movement was identical in all experiments, requiring subjects to make a 10-cm reaching movement from a central location (in the midsagittal plane ∼30 cm below the eyes and 30 cm in front of the chest) to one of eight equally spaced peripheral targets (Fig. 1*B*). During this movement, the vBOT was either passive (null field) or produced a clockwise (CW) or a counterclockwise (CCW) velocity-dependent curl field. For the velocity-dependent curl field (Gandolfo et al. 1996), the force at the handle was given by

$$\left[\begin{array}{c} F_{x}\\ F_{y} \end{array}\right]=k\left[\begin{array}{cc} 0 & -1\\ 1 & 0 \end{array}\right]\left[\begin{array}{c} \dot{x}\\ \dot{y} \end{array}\right]$$

where *k* was set equal to ±13 N/m·s. The sign of *k* determined the direction of the force field (CW or CCW), and this varied pseudorandomly from trial to trial.

One type of contextual cue was used in each experiment, and the direction of the field was predictable based on the contextual cue. The experiments were counterbalanced such that in each experiment, half of the subjects experienced the contextual cues matched to one set of force field directions, whereas the other half of the subjects experienced the contextual cues matched to the opposite force field directions. This was performed to avoid any bias arising from associations between a particular context and field direction. The ability of seven different contextual cues to reduce the interference between the two fields (CW and CCW) was examined.

Within each experiment, blocks of 18 trials were performed consisting of 16 field trials and 2 clamp trials. In the field trials, each of the eight targets was presented with both of the two possible field directions. The order of the movements within each block was pseudorandom, except that a clamp trial always occurred in the first and last four trials of each block. The two clamp trials always occurred for movements to the 0° target (straight ahead), one trial with a contextual cue for a CW field and one trial with a contextual cue for a CCW field (randomizing which came first within each block). In a clamp trial, the movement was confined to a simulated mechanical channel with a spring constant of 10,000 N/m (Milner and Franklin 2005; Scheidt et al. 2000; Smith et al. 2006).

Each experiment began with a preexposure phase consisting of 12 blocks in which no forces were applied (216 null trials), followed by an exposure phase of 75 blocks (1,350 field trials), and finally a postexposure phase consisting of 4 blocks (72 null trials). Subjects were given a short rest on average every 200 trials (195–205 trials).

On each trial, the central start location was displayed and the robotic manipulandum passively moved the subject's hand to the center location (following a minimum jerk trajectory). A trial was initiated by the hand cursor remaining within the home location at a speed below 0.1 cm/s for 300 ms. Both the target and the contextual cue were then presented, with the latter depending on (and therefore predictive of) the direction of the upcoming curl field (in the exposure condition). After a delay of 1,000 ms, an acoustic tone sounded to indicate that the subject should initiate the movement to the target (within 500 ms). Subjects were required to wait in the starting location for 1,000 ms preceding the acoustic signal to ensure that they had time to observe the contextual cue and also to ensure that prior passive movement to the starting location could have no contextual effect (Howard et al. 2012). If the duration of the movement (measured from the time the cursor had moved 2 cm from the center location until it entered the target) was between 150 and 250 ms, a “GREAT” message was displayed; otherwise a “TOO FAST” or a “TOO SLOW” warning was given accordingly.

#### Experiment 1: cursor color.

**Fig. 2.**
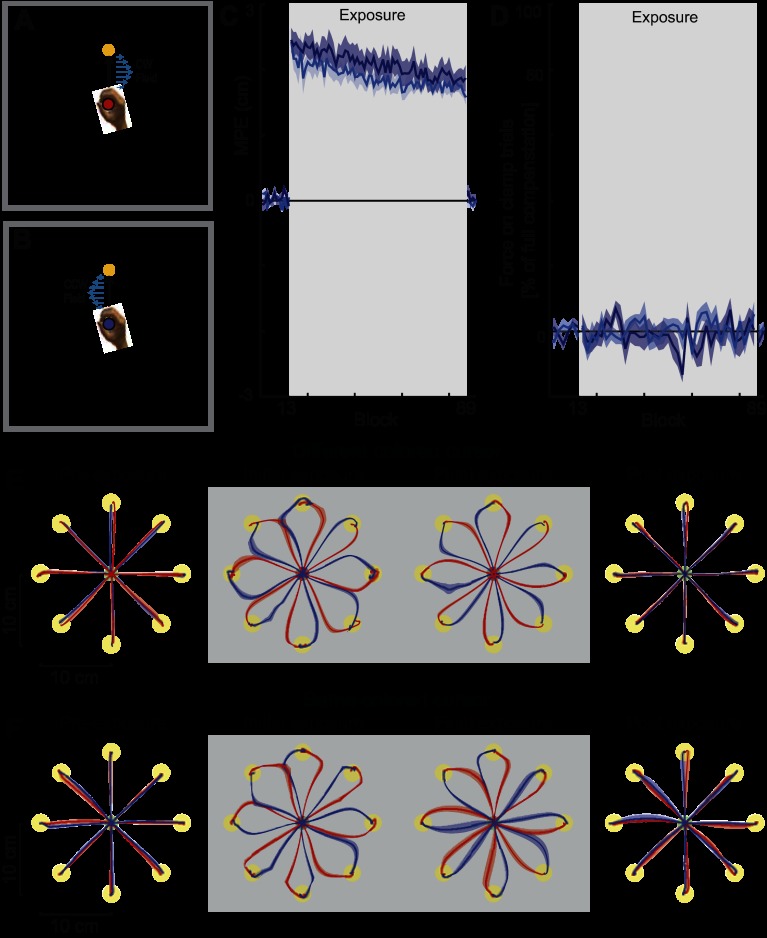
Cursor color and same color cursor. *A*: experimental design. Subjects started the trial with their hand at the central location while the target (yellow circle) was visually presented. Movement initiation is signified by an acoustic beep. In the cursor color experiment, the cue (red cursor) uniquely specified the clockwise (CW) force field applied once the subjects initiate the movement to the target (T1). *B*: for the same target (T1), the cue (blue cursor) uniquely specified the counterclockwise (CCW) force field, applied once the movement was initiated. The same cursor color experiment was performed in the same manner but with the same cursor color for both conditions. *C*: maximum perpendicular error (MPE) plotted against block number. The mean across all subjects (dark blue solid line for color cursor condition, light blue solid line for same cursor color condition) and standard error across subjects (with dark and light blue shaded regions, respectively) for each block in the experiment are shown. Although the 2 force fields produce error in the opposite directions, the sign of errors on trials on which the CCW field was presented have been reversed so that all errors in the direction of the force field are shown as positive. On *block 13*, the 2 curl fields were introduced (exposure, gray shaded region), which remained on until *block 89*, when subjects returned to the null force field. *D*: percent force compensation computed from clamp trials throughout the experiment. The mean force (±SE) across subjects over 2 batches is plotted as a percentage of the force required for estimated complete compensation. Shaded region indicates exposure blocks in which the curl force fields were applied. *E*: color cursor experiment hand paths during the movements between the central location and target (yellow circles). The mean (solid line), standard error (dark shaded region), and standard deviation (light shaded region) across all subjects for each condition are plotted. The trials for the context in which the CW force field was applied are shown in red, whereas the trials for the context in which the CCW force field was applied are shown in blue. Preexposure, the mean across subjects for 16 trials in the initial null field (*block 12*); initial exposure, the first 16 trials in the curl force fields (*block 13*); final exposure, the last 16 trials in the curl field (*block 88*); postexposure, the first 16 trials in the null field during the washout (aftereffect trials, *block 89*). Trials in which the force field is applied are shown with the shaded gray background. *F*: hand paths during the movements plotted as in *E* for the same cursor color condition.

*Experiment 1* (*n* = 6) examined the contextual effects of a colored cursor. On each trial, the color of the cursor was either red or blue, and this uniquely specified the direction of the associated curl field (2 field trials are shown in Fig. 2, *A* and *B*, for CW and CCW fields, respectively).

#### Experiment 2: same cursor color.

*Experiment 2* (*n* = 6) was identical to *experiment 1*, except the cursor color was always red. This provided a control condition in which no visual contextual information was available to the subjects.

#### Experiment 3: movement in different workspace locations.

**Fig. 3.**
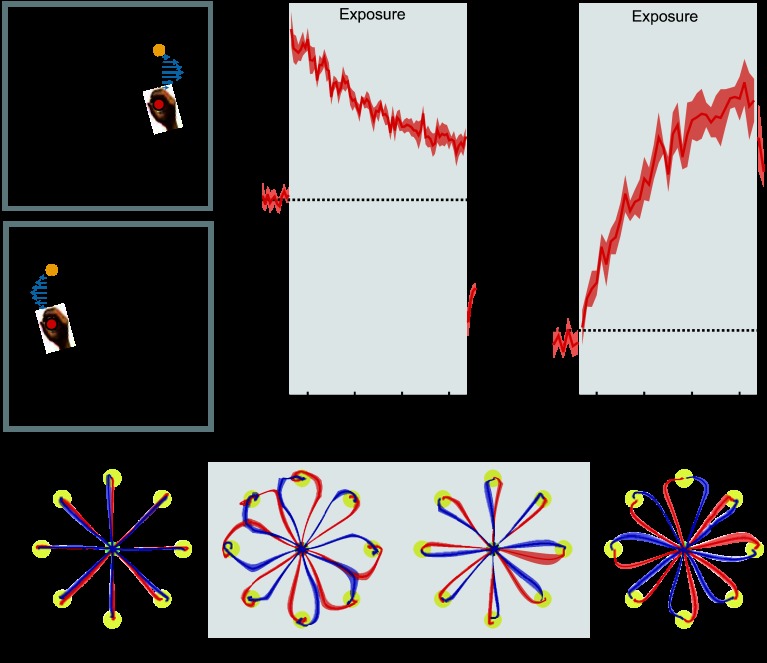
Movement in different workspace locations. Results are plotted as in Fig. 2. *A*: experimental design showing cues and associated field directions. The cue (task offset to the right) uniquely specified the CW force field, applied once the subjects initiate the movement to the target (T1). *B*: for the same target (T1), the cue (task offset to the left) uniquely specified the CCW force field, applied once the movement was initiated. *C*: mean MPE (red trace) and standard error (red shaded region) across all subjects, as a function of experimental block. *D*: percent force compensation in the colored cursor condition. *E*: mean and standard error of hand paths across subjects for the 16 trials during the movements between the central location and target (yellow circles) for single blocks during preexposure, initial exposure, final exposure, and postexposure.

*Experiment 3* (*n* = 6) examined the contextual effects of movements made in different workspace locations, with location determining the field direction (2 field trials are shown in Fig. 3, *A* and *B*, for CW and CCW fields, respectively). The center location was 10 cm to either the right or the left of the midsagittal plane.

#### Experiment 4: background frame color.

**Fig. 4.**
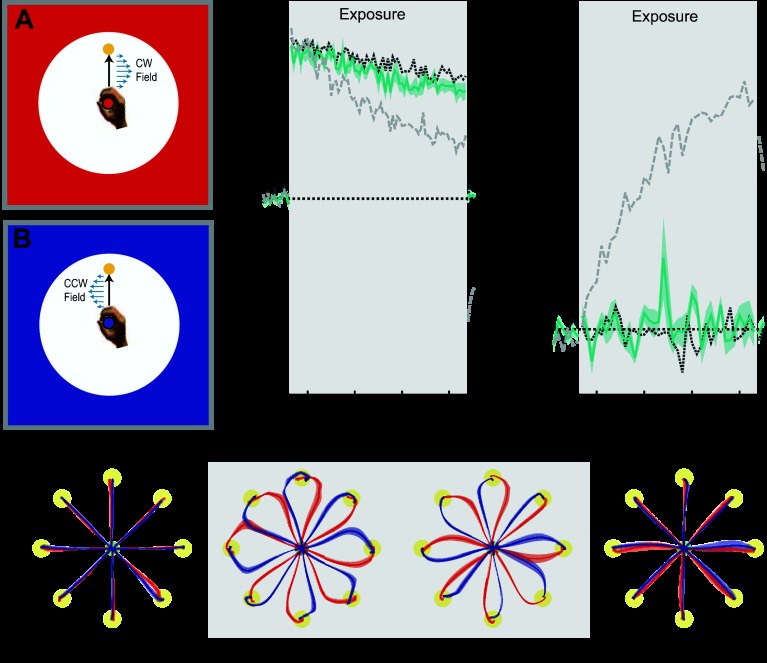
Background frame color. Results are plotted as in Fig. 2. In all conditions to aid comparison, the mean results for the colored cursor (dark gray dotted trace) and different workspace location conditions (light gray dashed trace) are shown on both MPE and force compensation plots. Shaded gray region indicates exposure period in which the 2 curl force fields were applied. *A* and *B*: experimental design showing cues and associated field directions. *C*: mean MPE (turquoise trace) and standard error (turquoise shaded region) across all subjects, as a function of experimental block. In this condition the color of the background frame (blue or red) surrounding the movement task signified context. *D*: percent force compensation in the colored frame condition. *E*: mean and standard error of hand paths across subjects for the 16 trials during the movements between the central location and target (yellow circles) for single blocks during preexposure, initial exposure, final exposure, and postexposure.

*Experiment 4* (*n* = 6) examined the contextual effects of background frame color. The background frame consisted of a rectangle that filled the display. To ensure the colored frame did not obscure the movements, a black circle (radius 13 cm) was centered on the central home position so that the targets could be overlaid. The entire background frame and cursor color was either red or blue, indicating the direction of the associated curl field (2 field trials are shown in Fig. 4, *A* and *B*, for CW and CCW fields, respectively).

#### Experiment 5: peripheral visual motion.

**Fig. 5.**
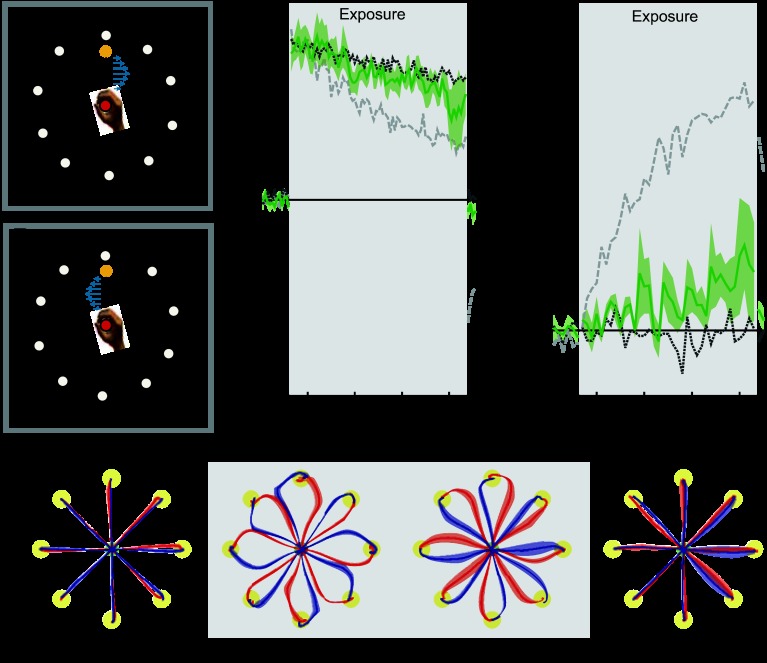
Peripheral visual movement. Results are plotted as in Fig. 2. In all conditions to aid comparison, the mean results for the colored cursor (dark gray dotted trace) and different workspace location conditions (light gray dashed trace) are shown on both MPE and force compensation plots. Shaded gray region indicates exposure period in which the 2 curl force fields were applied. *A* and *B*: experimental design showing cues and associated field directions. In this condition the direction of rotation of 10 disks around the periphery of the workspace (CW or CCW) signified context. *C*: mean MPE (green trace) and standard error (green shaded region) across all subjects, as a function of experimental block. *D*: percent force compensation in the unrelated movement condition. *E*: mean and standard error of hand paths across subjects for the 16 trials during the movements between the central location and target (yellow circles) for single blocks during preexposure, initial exposure, final exposure, and postexposure.

In *experiment 5* (*n* = 6) the contextual cue was provided by displaying 10 moving disks that rotated either clockwise or counterclockwise, depending on the direction of the associated curl field (2 field trials are shown in Fig. 5, *A* and *B*, for CW and CCW fields, respectively). The moving disks each had a radius of 0.5 cm and rotated at speed of 54°/s around the circumference of a circle centered on the home position of radius 14 cm.

#### Experiment 6: visual object orientation.

**Fig. 6.**
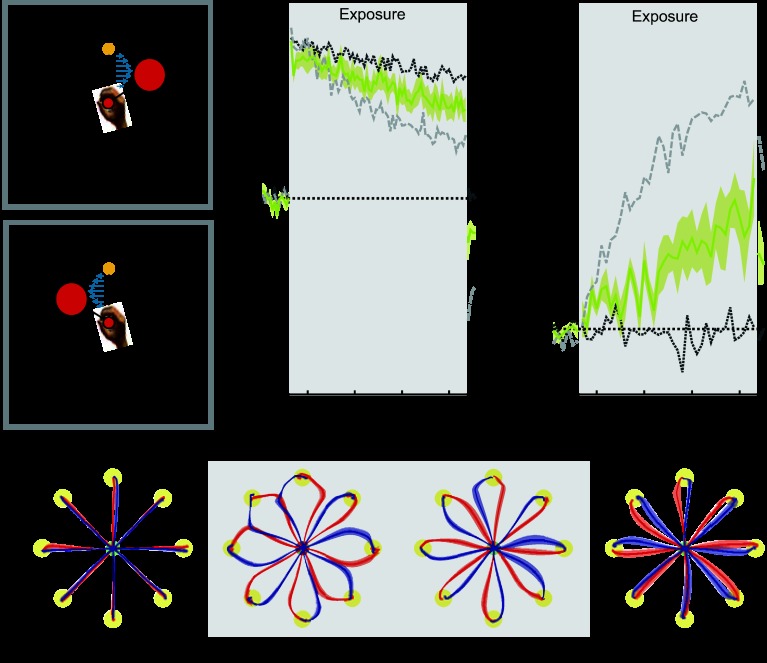
Visual object orientation. Results are plotted as in Fig. 2. In all conditions to aid comparison, the mean results for the colored cursor (dark gray dotted trace) and different workspace location conditions (light gray dashed trace) are shown on both MPE and force compensation plots. Shaded gray region indicates exposure period in which the 2 curl force fields were applied. *A* and *B*: experimental design showing cues and associated field directions. In this condition the orientation of an object attached to the cursor (left or right) task signified context. *C*: mean MPE (yellow trace) and standard error (yellow shaded region) across all subjects, as a function of experimental block. *D*: percent force compensation in the object orientation condition. *E*: mean and standard error of hand paths across subjects for the 16 trials during the movements between the central location and target (yellow circles) for single blocks during preexposure, initial exposure, final exposure, and postexposure.

*Experiment 6* (*n* = 6) examined whether the visual orientation of an object, such as a tool held in the hand, can act as a contextual cue. The red hand cursor was connected to a 2-cm radius red disk via a 10 × 0.2-cm red stick that was oriented to either the left or right of the cursor position (Fig. 6, *A* and *B*, for CW and CCW fields, respectively). The object was rotated 30° from the horizontal to avoid obscuring any targets or central home position.

We note that the direction of the visual object orientation, although informative as to whether subjects will experience a CW or CCW field, has no consistent relation to the direction of the forces that will be experienced. That is, the orientation of the object for one field is fixed, but the direction of the force experienced depends on the movement direction and hence which of the eight targets the subject is reaching to. Therefore, the direction of the force experienced across the eight movement directions spans all possible orientations (8 equally spaced over 360°) relative to the object orientation. In addition, the field direction is counterbalanced across subjects so that the subjects cannot rely on the use of any intuitive physical relation between the object orientation and expected force.

#### Experiment 7: visual feedback location.

**Fig. 7.**
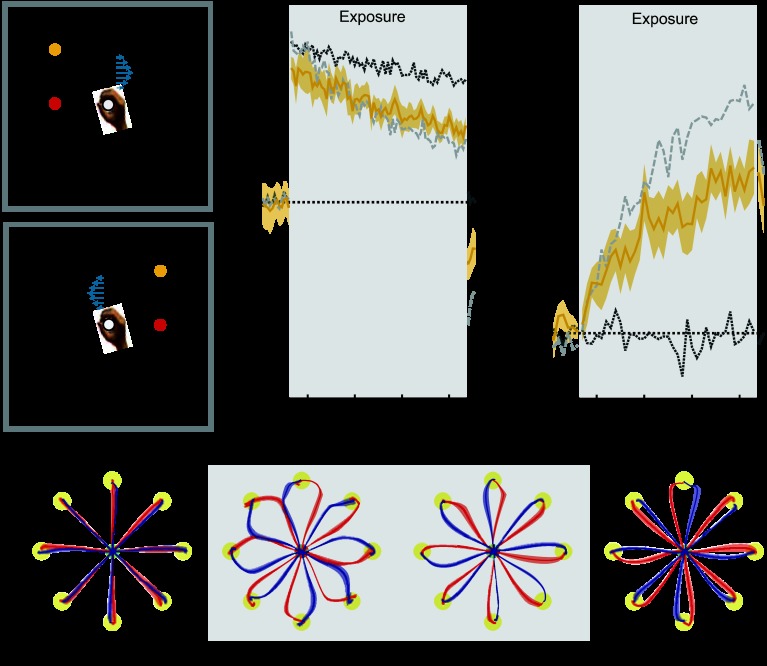
Visual feedback location. Results are plotted as in Fig. 2. In all conditions to aid comparison, the mean results for the colored cursor (dark gray dotted trace) and different workspace location conditions (light gray dashed trace) are shown on both MPE and force compensation plots. Shaded gray region indicates exposure period in which the 2 curl force fields were applied. *A* and *B*: experimental design showing cues and associated field directions. In this condition the visual appearance of the task shifted right or left from the central position by 10 cm signified context, but the movement always took place centrally. *C*: mean MPE (light orange trace) and standard error (light orange shaded region) across all subjects, as a function of experimental block. *D*: percent force compensation in the visual shift condition. *E*: mean and standard error of hand paths across subjects for the 16 trials during the movements between the central location and target (yellow circles) for single blocks during preexposure, initial exposure, final exposure, and postexposure.

*Experiment 7* (*n* = 6) examined the contextual effects of visual feedback being provided in different workspace locations. Movements were always made in the same central location, but the entire visual feedback (center location, targets, and cursor) was offset by 10 cm to the left or to the right of midline, with the direction of offset determining the field direction (Fig. 7, *A* and *B*, for CW and CCW fields, respectively). As in the visual object orientation experiment, we note that the direction of the visual shift, although informative as to whether subjects will experience a CW or CCW field, has no consistent relation to the direction of the forces experienced.

#### Experiment 8: proprioceptive location.

**Fig. 8.**
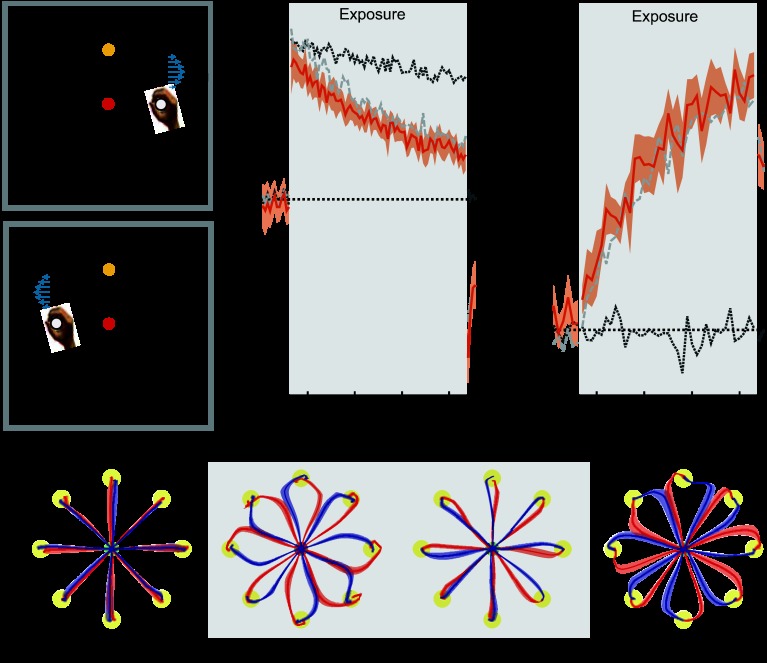
Proprioceptive location. Results are plotted as in Fig. 2. In all conditions to aid comparison, the mean results for the colored cursor (dark gray dotted trace) and different workspace location conditions (light gray dashed trace) are shown on both MPE and force compensation plots. Shaded gray region indicates exposure period in which the 2 curl force fields were applied. *A* and *B*: experimental design showing cues and associated field directions. In this condition the physical location of the movement from the central position signified context, but the task was always displayed (i.e., home position, cursor location, and target position) in the center position. *C*: mean MPE (dark orange trace) and standard error (dark orange shaded region) across all subjects, as a function of experimental block. *D*: percent force compensation in the proprioceptive shift condition. *E*: mean and standard error of hand paths across subjects for the 16 trials during the movements between the central location and target (yellow circles) for single blocks during preexposure, initial exposure, final exposure, and postexposure.

*Experiment 8* (*n* = 6) examined the contextual effects of movements being made in different workspace locations. The entire visual feedback (center location, targets, and cursor) was always displayed in the same central location, but the movements were offset by 10 cm to the left or to the right of midline (by introducing the appropriate offset between the robot handle location and the displayed hand cursor), with the direction of offset determining the field direction (Fig. 8, *A* and *B*, for CW and CCW fields, respectively).

#### Data analysis.

Data were collected from the manipulandum encoders and force transducer at 1,000 Hz and logged to disk for off-line analysis using MATLAB (The MathWorks, Natick, MA). Movement error was calculated on each trial by analyzing the hand movement from the central to the target location. The maximum perpendicular error (MPE) of the hand path from a straight path from the center location to the target was calculated. For each subject, the MPE was sign adjusted appropriately so that errors from CW and CCW curl field trials could be combined, and then the average of the MPE for all exposure trials within a block was computed. The mean and SE for each block across subjects was then calculated.

The endpoint forces were examined on the clamp trials to further quantify the amount of adaptation that occurred in the experiments. The force produced by subjects into the wall of the simulated channel was integrated across the movement. To evaluate the degree of compensation, the measured force was divided by the amount of force that would be required for perfect compensation in the force field (calculated as the field constant multiplied by the actual velocity integrated across the movement on each trial). The values of percent force compensation throughout the experiment are based on the compensation required in the curl force field. Therefore, values in the null force field before learning (preexposure phase) should be close to zero.

We performed hypothesis-based planned comparisons and report uncorrected *P* values to determine statistical significance. Statistical differences were determined with an ANOVA in SPSS 21.0 using the general linear model. A general linear model was used to examine if the force field produced significant changes in movement error during the initial exposure phase (MPE on first 4 blocks; *blocks 13–16*) compared with the initial null field exposure (final 4 blocks; *blocks 9–12*), with a factor of experiment (8 levels). For all experiments, a general linear model was used to test if the error was significantly different at the end of exposure (MPE on last 4 exposure blocks; *blocks 84–87*) compared with initial exposure (MPE on first 4 exposure blocks; *blocks 13–16*), with subjects as a random effect. A second linear model was used to test if there were significant aftereffects (MPE on all 4 blocks in the final null field; *blocks 88–91*) compared with the initial null field trials (MPE on last 4 blocks in the null field; *blocks 9–12*), with subjects as a random effect. Similar tests were also performed on percent force compensation where appropriate. Statistical significance was considered at the *P* < 0.05 level for all statistical tests.

## RESULTS

In all experimental conditions, subjects performed trials with contextual cues present both before and during reaching. The two contexts uniquely specified the direction of a velocity-dependent curl field. Subjects performed blocks of 18 trials presented in a pseudorandom order, 2 of which were clamp trials. Each experiment began with 12 blocks in the null field, followed by 75 field exposure blocks and finally 4 blocks in the null field. Across all experiments and subjects, the peak displacement into the channel on clamp trials was 0.36 (SD 0.18) mm.

In all experiments, subjects initially performed movements in the null field, making straight movements to each of the eight targets, regardless of the cue context (Fig. 2, *E* and *F*, and Figs. 3*E*–8*E*, preexposure). When the force fields were introduced, the initial movements showed large deviations from a straight line, in the direction of the force field (Fig. 2, *E* and *F*, and Figs. 3*E*–8*E*, initial exposure). To assess whether the introduction of the force field led to similar levels of kinematic error across the eight conditions, we performed an ANOVA on MPE with factors of exposure period (2 levels: last block preexposure and first block initial exposure) and experimental condition (8 levels). There was a significant increase in MPE during the initial exposure period across all seven experimental conditions (main-effect exposure period: *F*_1,368_ = 2,688.677; *P* < 0.001), with no significant differences in the size of the induced perturbations across all conditions (interaction effect between experimental condition and exposure period: *F*_7,368_ = 1.185; *P* = 0.310). However, the forms of the trajectories in the final exposure and postexposure blocks depended on the effectiveness of each individual contextual cue condition and were analyzed separately.

### 

#### Experiment 1: cursor color.

During *experiment 1* the cursor color (red or blue) was uniquely associated with one of the two curl field directions (directions counterbalanced across subjects). We quantified performance by the MPE on each trial (Fig. 2*C*, dark blue) and also the amount of force compensation as determined by the force produced against the channel wall on clamp trials (Fig. 2*D*, dark blue). Although over the course of the exposure period MPE decreased (Fig. 2*E*, final exposure; *F*_1,5_ = 58.220; *P* = 0.001), no significant increase in force compensation was seen (*F*_1,5_ = 0.223; *P* = 0.657) from the initial to the final trials of the exposure period. Moreover, on removal of the field during the postexposure period, no deviation of trajectories from a straight line were observed, indicating that subjects had not learned a specific compensation for the two opposing force fields (Fig. 2*E*, postexposure). An ANOVA confirmed that there was no significant difference in MPE (*F*_1,5_ = 0.00; *P* = 0.994) or percent force compensation (*F*_1,5_ = 1.896; *P* = 0.227) between the null field trials of the postexposure and preexposure periods.

The small but significant decreases in MPE during field exposure, taken in isolation, could be interpreted as subjects learning a small amount of compensation for the force fields. However, the absence of aftereffects (significant deviations of the trajectory during the postexposure phase) suggests no field-specific compensation. Instead, these results imply that the reduction in MPE during the exposure period is likely to have occurred through a field-independent increase in limb stiffness (Burdet et al. 2001; Franklin et al. 2007; Takahashi et al. 2001), driven by increases in muscular cocontraction (Franklin et al. 2003; Milner and Cloutier 1998; Osu et al. 2002) and feedback gains (Franklin et al. 2012). Overall, these findings demonstrate that, despite the reduction in MPE during the experiment, cursor color does not provide contextual information suitable for learning opposing force fields over the time course of our paradigm.

#### Experiment 2: same cursor color.

*Experiment 2* provided a control condition and examined subject behavior when no visual contextual information was available to the subjects. Over the exposure period the MPE decreased slightly (*F*_1,5_ = 12.560; *P* = 0.016), whereas no significant increase in force compensation was found (*F*_1,5_ = 1.623; *P* = 0.259) from the initial to the final trials of the exposure period (Fig. 2, *C* and *D*, light blue traces). Moreover, on removal of the field during the postexposure period, no deviation of trajectories from a straight line were observed, indicating that subjects had not learned a specific compensation for the two opposing force field (Fig. 2*F*, postexposure). An ANOVA confirmed that there was no significant difference in MPE (*F*_1,5_ = 0.01; *P* = 0.925) or percent force compensation (*F*_1,5_ = 0.001; *P* = 0.976) between the null field trials of the postexposure and preexposure periods.

In this condition, there is no contextual information available to the subjects from the appearance of the cursor, and the absence of channel force in the channel trials indeed indicates no learning took place. However, just as in *experiment 1*, there was a slight reduction in MPE during the exposure phase. The striking similarity in results from these two experiments provides strong support for the hypothesis that the observed reduction in MPE during the color cursor (*experiment 1*) arose from cocontraction, allowing us to confirm that a colored cursor is an ineffective contextual cue.

#### Experiment 3: movement in different workspace locations.

In *experiment 3*, movements were made in different workspace locations, with location determining the field direction. Over the course of the exposure period, trajectory deviations produced by the introduction of the curl force field diminished substantially (Fig. 3*E*, final exposure). Using an ANOVA, we found a significant reduction in MPE from the initial to the final trials of the exposure period (*F*_1,5_ = 82.295; *P* < 0.001). At the same time we found a significant increase in the endpoint force that was produced against the channel on the clamp trials (*F*_1,5_ = 55.284; *P* = 0.001). On removal of the field during the postexposure period, very strong deviations of trajectories from a straight line in the opposite direction were observed, indicating that subjects had learned a specific compensation for the two field directions (Fig. 3*E*, postexposure). During these postexposure null field trials, an ANOVA found both a significantly increased MPE (*F*_1,5_ = 168.425; *P* < 0.001) and force compensation (*F*_1,5_ = 148.237; *P* < 0.001) compared with the preexposure period. Overall, these findings demonstrate that movement in different parts of the workspace associated with different curl field direction allows substantial learning of opposing curl force fields.

The results of both *experiment 1* (color cue), in which no learning was found, and *experiment 3* (workspace location), in which strong learning was found, provide baselines for the interpretation and comparison of all remaining experimental conditions. To facilitate comparison across conditions, the MPE and force compensation results for these two experiments are included in subsequent Figs. 4–8.

#### Experiment 4: background frame color.

*Experiment 4* examined the contextual effect of background frame color. On each trial, cursor and background color (red or blue) provided information indicating the direction of the curl force field presented on that trial during the exposure phase.

Over the course of the exposure period, trajectory deviations induced by the curl force fields were still prominent (Fig. 4*E*, final exposure). Using an ANOVA, we found a small but significant reduction in MPE (*F*_1,5_ = 13.282; *P* = 0.015) but no significant increase in the percentage force compensation (*F*_1,5_ = 0.78; *P* = 0.791) from the initial to the final trials of the exposure period. On removal of the field during the postexposure period, no deviation of trajectories from a straight line were observed, indicating that subjects did not learn specific compensation for the two field directions (Fig. 4*E*, postexposure) and that any reduction in MPE is likely attributable to nonspecific changes in stiffness.

Supporting this interpretation, both MPE (*F*_1,5_ = 0.354; *P* = 0.578) and force compensation (*F*_1,5_ = 0.514; *P* = 0.506) on the null field trials of the postexposure period were not significantly different from those of the preexposure period. Overall, the findings demonstrate that background frame color cues, even when they are visually striking, provide little or no contextual information that is suitable for learning opposing force fields.

#### Experiment 5: peripheral visual motion.

*Experiment 5* examined the contextual effect of peripheral visual motion. On each trial, the direction of rotation of a set of white disks provided contextual information.

Over the course of the exposure period, deviations from a straight line diminished slightly (Fig. 5*E*, final exposure). Using an ANOVA, we found no significant reduction in MPE from the initial to the final trials of the exposure period (*F*_1,5_ = 2.252; *P* = 0.194) and no increase in force compensation (*F*_1,5_ = 0.784; *P* = 0.416) during the same trials. On removal of the field during the postexposure period, small deviations of trajectories from the straight line were observed (Fig. 5*E*, postexposure). However, neither the MPE (*F*_1,5_ = 1.066; *P* = 0.349) nor force compensation (*F*_1,5_ = 3.911; *P* = 0.105) on the null postexposure trials were significantly different from those of the preexposure period. Overall, these findings demonstrate that peripheral visual motion cues do not allow the learning of opposing force fields with the timescales used in this experiment.

#### Experiment 6: visual object orientation.

*Experiment 6* examined the contextual effect of the orientation of a visual object attached to the cursor. Over the course of the exposure period, deviations from a straight line reduced slightly (Fig. 6*E*, final exposure). Using an ANOVA, we found a significant reduction in MPE (*F*_1,5_ = 10.802; *P* = 0.022) and a significant increase in force compensation (*F*_1,5_ = 18.678; *P* = 0.008) from the initial to the final trials of the exposure period. On removal of the field during the postexposure period, some deviation of trajectories from a straight line were observed, indicating that subjects learned a specific compensation for the two field directions (Fig. 6*E*, postexposure). During the null field trials of the postexposure period, MPE (*F*_1,5_ = 10.820; *P* = 0.022) and force compensation (*F*_1,5_ = 7.336; *P* = 0.042) were significantly different from those of the preexposure period. Overall, these findings demonstrate that object orientation cues provide contextual information suitable for some learning of the opposing force fields.

#### Experiment 7: visual feedback location.

In *experiment 7*, movements were always made in the same central location, but the entire visual feedback (center location, targets and cursor) was offset to the left or to the right of midline, with the direction of offset determining the field direction.

Over the course of the exposure period, deviations from a straight-line diminished substantially (Fig. 7*E*, final exposure). Using an ANOVA, we found a significant reduction in MPE (*F*_1,5_ = 44.386; *P* = 0.001) and a significant increase in force compensation (*F*_1,5_ = 34.638; *P* = 0.002) from the initial to the final trials of the exposure period (Fig. 7, *C* and *D*). On removal of the field during the postexposure period, strong deviation of trajectories from a straight line were observed, indicating that subjects learned a specific compensation for the two curl field directions (Fig. 7*E*, postexposure). Correspondingly, both the MPE (*F*_1,5_ = 86.718; *P* < 0.001) and the force compensation (*F*_1,5_ = 34.742; *P* = 0.002) on the postexposure trials were significantly larger than on the preexposure trials. Overall, these findings demonstrate that the location of visual feedback of the movements provides strong contextual information that is suitable for learning opposing force fields.

#### Experiment 8: proprioceptive location.

In *experiment 8*, the entire visual feedback was always displayed in the same central location, but the movements were offset to the left or to the right of midline, with the direction of offset determining the field direction.

Over the course of the exposure period, deviations from a straight line diminished substantially (Fig. 8*E*, final exposure). Using an ANOVA, we found a significant reduction in MPE (*F*_1,5_ = 77.447; *P* < 0.001) and a significant increase in force compensation (*F*_1,5_ = 197.048; *P* < 0.001) from the initial to the final trials of the exposure period. On removal of the field during the postexposure period, strong deviation of trajectories from a straight line were observed, indicating that subjects had learned a specific compensation for the two field directions (Fig. 8*E*, postexposure). Correspondingly, both the MPE (*F*_1,5_ = 123.717; *P* < 0.001) and the force compensation (*F*_1,5_ = 177.681; *P* < 0.001) on the postexposure trials were significantly larger than on the preexposure trials. Overall, these findings demonstrate that differences in proprioceptive location without any change in visual feedback of the task provide strong contextual information that is suitable for learning opposing force fields.

#### Comparison of contextual effects across conditions.

**Fig. 9.**
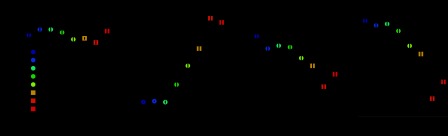
Contextual effects as a function of cue. *A*: mean velocity (±SE) on clamp trials (in direction toward target T1). Values for each experimental condition [on last third of trials (25 blocks) during force field exposure] are plotted sequentially along the *x*-axis. *B*: mean force (±SE) on clamp trials (in direction toward target T1) as a percentage of the force required for estimated complete compensation. Values for each experimental condition [on last third of trials (25 blocks) during force field exposure] are plotted sequentially along the *x*-axis. *C*: mean MPE (±SE) over the last block of trials during force field exposure. *D*: mean MPE (±SE) over the first block of trials in the postexposure session in all experiments.

In each experiment, a clamp trial (toward the straight-ahead target) for each contextual cue was presented in each block to examine feedforward adaptation. The degree of force compensation that occurred to the force field was examined on these trials over the last third of the blocks during the force field exposure. Although the velocities in these clamp trials were similar across all conditions (Fig. 9*A*), the amount of force compensation varied across the conditions (Fig. 9*B*). An ANOVA (with main factors of block number and experimental condition) indicated a significant main effect of experimental condition (*F*_7,1000_ = 268.954; *P* < 0.001), and this was further examined using post hoc tests. There was no significant difference in the amount of force compensation for the same cursor color condition, the color cursor condition, or the visual frame condition (all *P* > 0.986). Similarly, there also was no difference in the amount of force compensation between the proprioceptive location and the workspace shift (*P* = 0.862). However, there were significant differences between all other experimental conditions (all *P* < 0.001). Only for the workspace shift and physical shift conditions did the force field compensation over the last third of the exposure period approach 80% (Fig. 9*B*).

The percentage of force compensation varied greatly across the experimental conditions. This level of adaptation measured on clamp trials during the exposure period was also mirrored by MPE, both in the final exposure period (Fig. 9*C*) and in the postexposure aftereffects (Fig. 9*D*). In both of these measures, using only the last block of trials in a single-factor ANOVA, significant main effects of experimental condition (final exposure MPE: *F*_7,40_ = 4.355; *P* = 0.001; postexposure MPE: *F*_7,40_ = 21.613; *P* < 0.001) were found, indicating differences across these experimental conditions.

## DISCUSSION

Previous studies have demonstrated that there is a link between sensory information and the ability to learn and recall motor patterns, with some contextual cues being more effective than others. However, these studies examined a single or a small set of cues, each often using a unique paradigm, with different task parameters and movement numbers, making quantitative comparisons between the results difficult. In the present study we used an interference task to undertake a systematic investigation of the contextual effects of seven cues along with a pure control condition. This enabled us to quantify them in terms their ability to form distinct motor memories to opposing dynamics. A specific contextual cue was associated with each of two opposing force fields. The amount of learning was examined by using the reduction in kinematic error and increase in force compensation during the exposure trials, as well as the size of aftereffects (in both kinematic error and force) during the postexposure trials. We found dramatic differences in the efficacy of these sensory contextual cues. Our results show that although some visual cues, such as color, have little or no effect, other cues, such as changes in spatial visual and proprioceptive states of the movement, are far more effective and can lead to the formation and recall of distinct representations in motor memory.

The results demonstrated that changes in color (of either the cursor or background), while able to slightly but significantly reduce kinematic error during the field exposure, did not result in changes in endpoint force or aftereffects. Importantly, a control experiment, in which an identical red cursor indicated both curl field directions (therefore providing no visual contextual information), provided identical results. That is, a slight reduction in MPE was observed during field exposure, with no accompanying changes in endpoint force or aftereffects. Although reduction in kinematic error is often assumed to indicate adaptation to dynamics, our results indicate that subjects did not form a force field-specific motor memory of the two dynamics, since the latter would have produced force compensation and aftereffects. Instead, the results suggest that subjects used a general strategy (contextually nonspecific) of increasing limb stiffness (Burdet et al. 2001; Franklin et al. 2007; Takahashi et al. 2001) through increases in cocontraction (Franklin et al. 2003; Milner and Cloutier 1998; Osu et al. 2002) or feedback gains (Franklin et al. 2012). Such changes will reduce the perturbing effects of the force field but produce no net force on the channel wall or aftereffects when the force fields are removed. Therefore, on the timescale of our experiment, cursor color had essentially no effect on reducing interference. These results are consistent with previous studies that have indicated no learning (Gandolfo et al. 1996) and with others that have shown substantial training is required for a color to have an effective contextual effect (Krouchev and Kalaska 2003; Wada et al. 2003).

Despite its strong salient appearance, we found no evidence for frame color acting as contextual cue, and we were unable to reproduce the learning effects that have been previously reported (Osu et al. 2004). Although significant decreases in error occurred in both our study and that of Osu et al. (2004), here we have shown that decreases in endpoint error are not always associated with contextually appropriate adaptation of endpoint forces. We note that a previous study has shown a strong and rapid contextual effect of color during dynamic learning in human participants (Addou et al. 2011). There are several differences in the task and perturbation used in this previous study that could explain the observed learning. First, their task involved control of a single degree of freedom; that is, elbow flexion and extension. Second, only two targets were used, a flexion and an extension target from a central start location. Third, the applied perturbations were purely resistive and assistive viscous forces. In contrast, in our experiments we applied two-dimensional force fields during unconstrained planar (2 joint) movements to eight targets. We hypothesize that the ability to learn to switch on the basis of color described by Addou et al. (2011) arises because of the simplicity of the field and task. In particular, when the number of movements is small and the perturbation simple, it is possible to employ more cognitive strategies to achieve color-dependent switching. Our experiments were designed to hinder the use of cognitive strategies by employing complex fields and eight target directions. Our results clearly indicate that for such complex fields and multiple movement directions, color is, at best, a weak contextual cue, compared with other sensory contextual cues.

Although many studies have shown that static visual cues, such as cursor color (Gandolfo et al. 1996) or cue location (Howard et al. 2012), produce little or no contextual learning, recent work has shown that prior visual motion can act as a strong contextual cue (Howard et al. 2012). However, in this previous study, the visual motion was motion of the subject's cursor directly prior to movement in the force field. The direction of the cursor movement was predictive of the directionality of the force field experienced in the subsequent movement. From the current work it can be seen that an arbitrary visual motion cue does not produce such contextual effects even when it is uniquely associated with the force field direction. Here we investigated whether peripheral background movement within the workspace could act as a contextual cue by employing movement of disks around the periphery of the workspace. Such motion did not lead to context-dependent learning. Together, these results suggest that visual movement needs to be more closely associated with the task, or even be considered part of it by the motor system, for it to have a strong contextual effect. It remains to be determined exactly how closely associated the visual motion needs to be to the task, but it is clear that any arbitrary motion cannot be used to learn independent motor memories.

Previous studies have shown that subjects can use visual information to identify the location of the center of mass of objects (Bingham and Muchisky 1993). Subjects can also scale grip force to lift objects on the basis of visual appearance of objects (Jenmalm and Johansson 1997; Mon-Williams and Murray 2000; Wing and Lederman 1998). More recently, it has been shown that the visual appearance of a hammer at different orientations can be used to immediately recall the structural form of its complex grasp-dependent dynamics (Ingram et al. 2010). Therefore, there are a large number of studies demonstrating that the sensorimotor control system uses visual information of objects to determine the appropriate motor memory to use for control. Such studies motivated us to examine whether the addition of a visual object connected to the cursor could be associated with different dynamics. In particular we chose two arbitrary objects, matched in size, with no direct relation with the associated forces with either force field (considering all 8 directions of motion). Using this arbitrary association, we demonstrated that linking the field direction to the orientation of a visual object attached to the cursor significantly reduced interference, albeit by a small amount. Thus the orientation of an asymmetric cursor has a small but significant contextual effect, suggesting that the visual representation of an object can have a direct effect on the formation and recall of motor memories. However, we note that the asymmetric cursor condition exhibits some similarity to the visual offset condition, although its effects are much weaker. In both of these experiments the location of visual feedback was context dependent and offset to either the left or the right of the central home position. Thus, in the object orientation experiment, there may also exhibit some contextual effect due to differences in visual state of the cursor.

It has been suggested that the learning of dynamics occurs by representing the command necessary to compensate for the dynamics as a function of the state of the arm (Shadmehr and Mussa-Ivaldi 1994). This would suggest that learning at one location in the workspace might not interfere with learning at another location in the workspace if the movements were sufficiently different in the state space. Indeed, it has been shown that two opposing force fields can be learned independently when they are performed in single movements separated by more than 7 cm (Hwang et al. 2003). In our study, when subjects adapted to the two oppositely rotated curl force fields in two distinct workspace locations, little interference was observed and the predictive force compensation to the field directions as determined on the clamp trials approached 80%. This is at a level only slightly below what has been seen for single force field adaptation for either two (Joiner and Smith 2008) or four training directions (Howard et al. 2012). Therefore, these results support the findings of previous work that demonstrated little or no interference for force fields learned in different workspace locations provided there is sufficient distance between the workspace locations.

Learning two force fields in two different locations can be decomposed into two components. The first is that the visual feedback occurs in a different workspace, and the second is that the physical location (and proprioceptive feedback) of the movement occurs in different locations. When the individual effect of these two components were examined for single movement directions, it was found that learning was higher for differences in proprioception than for differences in vision, both of which were less than for the different workspace condition (Hwang et al. 2006). However, even for learning in the single direction of movement, the overall levels of adaptation were small. In our study we found significant reduction in interference for the shift in both visual and proprioceptive task location. Interestingly, in this case we found no difference in the degree of learning between the shift in proprioceptive task space and the shift in the workspace location. In contrast, when only the visual task location was different for the two fields, there was significantly more interference between the two tasks. The combined visual and proprioceptive shift is a state change, and the purely visual or purely proprioceptive shifts could be interpreted as either a cue or state change. We hypothesize that these are more likely to relate to changes in perceived state. Of course, we acknowledge that there are other differences that arise when the visual locations are separated, such as the direction of the subject's gaze, which could also act as a contextual cue.

The contextual effect of visual target shift in the absence of physical shift has also recently been studied through the introduction of visuomotor rotations (Hirashima and Nozaki 2012). Subjects made movements to two targets that were initially located 30° off the midsagittal line to the left and right, respectively. A corresponding 30° visuomotor rotation was gradually introduced to the two targets such that, after adaptation, subjects made identical physical movements along the midsagittal line for both targets. At this point, opposing curl fields associated with the two targets were introduced, to which subjects were able to adapt. Our results are consistent with these finding; both studies demonstrate significant learning but the presence of some interference limiting full compensation for the two force fields. However, our experimental results show that learning of opposing dynamics on the basis of contextual cues in the form of visual offsets can be achieved in multiple movement directions and without the need for a gradual introduction of the visual offset.

Overall, our findings indicate that more abstract aspects of vision, such as color, have a weaker, if any, effect on dynamic learning in the motor system than other visual aspects of the environment (such as object orientation), which may correlate more strongly with required changes in motor behavior. However, the strongest effects in this study were found for conditions in which the state space was sufficiently different between the two fields (through either visual or proprioceptive differences in state). We suggest that this difference may arise not simply because the different workspace cues are more salient, but because the motor memories are learned as a function of the state of the limbs. Because both visual and proprioceptive information can be used by the sensorimotor control system to estimate the limb state, distinct sensory cues from visual and proprioceptive modalities will influence the state space in which the novel dynamics are learned. We propose therefore that these differences illustrate a conceptual difference between two types of cue. First, subjects can learn to associate arbitrary sensory cues (e.g., object orientation in *experiment 5*) with different dynamics, but this effect takes substantial time and appears a weak cue. Second, sensory cues that dissociate the visual or proprioceptive state of the arm may automatically alter the neural coding of the movement so as to allow separate motor memories to be learned rapidly. We hypothesize that this distinction arises because dynamics can be represented in terms of the state of the limb, and modification of the proprioceptive or visual feedback of the limb will alter the estimated state. In contrast, other cues require associative learning to map the sensory cue with the appropriate internal model of the dynamics for both recall (before movement) and error assignment (during and after the motion) to produce an effect. This is because they have little a priori influence to the motor system. Indeed, the size weight illusion demonstrates this: it can be reversed, but only with substantial training (Flanagan et al. 2008).

In conclusion, we have shown that sensory contextual information can play a fundamental role in reducing interference and lead to the formation of separate motor memories for distinct dynamics. Our study has enabled us to rank the effectiveness of seven such contextual cues by using a standardized protocol across all experimental conditions. The results demonstrate that different aspects of sensory information provide better contextual cues than others, with conditions that could influence the estimated limb state having the strongest effects. Overall, this work demonstrates the importance of state-dependent sensory information in the formation and recall of independent motor memories.

## GRANTS

We thank the Wellcome Trust and the Human Frontiers Science Program for support.

## DISCLOSURES

The authors declare that they have no financial, personal, or professional interests that could be construed to have influenced the paper.

## AUTHOR CONTRIBUTIONS

I.S.H., D.M.W., and D.W.F. conception and design of research; I.S.H. performed experiments; I.S.H. and D.W.F. analyzed data; I.S.H., D.M.W., and D.W.F. interpreted results of experiments; I.S.H. and D.W.F. prepared figures; I.S.H. drafted manuscript; I.S.H., D.M.W., and D.W.F. edited and revised manuscript; I.S.H., D.M.W., and D.W.F. approved final version of manuscript.
